# Decision regret after external beam radiotherapy and high dose-rate brachytherapy boost for prostate cancer

**DOI:** 10.1007/s00345-025-05615-3

**Published:** 2025-04-09

**Authors:** Lars Haack, David Krug, Severin Rodler, Philipp Nuhn, Christof van der Horst, Christian Schulz, Olaf Wittenstein, Claudia Schmalz, Oliver Blanck, Frank-André Siebert, Alexander Fabian

**Affiliations:** 1 Department of Radiation Oncology, University Hospital Schleswig-Holstein Campus Kiel, Arnold-Heller-Str.3, 24105 Kiel, Germany; 2 Department of Radiotherapy and Radiation Oncology, University Medical Center Hamburg-Eppendorf, Hamburg, Germany; 3 Department of Urology, University Hospital Schleswig-Holstein Campus Kiel, Kiel, Germany; 4 URODOCK Urology Group Practice, Kiel, Germany

**Keywords:** Prostate cancer, Radiotherapy, Brachytherapy, Decision regret, Patient-reported outcome, Health-related quality of life

## Abstract

**Purpose:**

Patients with localized prostate cancer have various treatment options. Decision regret from a patient’s view is an unpleasant feeling concerning a decision in health care. We aimed to explore regret and its determinants after external beam radiotherapy (EBRT) with a high dose-rate brachytherapy (HDR-BT) boost as traditional method of treatment intensification.

**Methods:**

We conducted a secondary analysis of a cross-sectional study. Patients were enrolled at least two years after EBRT + HDR-BT. Decision regret was measured using the Decision Regret Scale (0–100). Covariables included patient characteristics, dose to organs at risk and patient-reported outcomes (PRO) including EPIC- 26, EORTC QLQ-C30, and PSCC. We used Pearson’s correlation and one-way ANOVA analyses.

**Results:**

Among 108 patients, the median age was 73 and the median interval since treatment was 4 years. The mean score of decision regret was 11 (SD: 14). No (0), mild (1–25), or strong (> 25) decision regret was present in 49% (53/108), 39% (42/108), and 12% (13/108) of the patients, respectively. PRO were associated with stronger decision regret including worse urinary incontinence (r = − 0.198; p = 0.046), worse urinary irritative/obstructive symptoms (r = − 0.203; p = 0.045), worse bowel function (r = − 0.312; p = 0.001), worse hormonal function (r = − 0.289; p = 0.003), lower levels of shared-decision making (r = − 0.292; p = 0.002), and lower patient satisfaction (r = − 0.326; p < 0.001).

**Conclusion:**

Long-term decision regret is mild among patients with prostate cancer treated with EBRT + HDR-BT. PRO were associated with decision regret which should be respected in clinical practice.

**Trial registration:**

The study protocol was registered prior to patient accrual on the Open Science Framework (doi.org/10.17605/OSF.IO/A6DC3).

**Supplementary Information:**

The online version contains supplementary material available at 10.1007/s00345-025-05615-3.

## Introduction

Localized prostate cancer is the leading cancer diagnosis among men in Western countries [1]. Treatment options are manifold and include surgery, external beam radiotherapy (EBRT), brachytherapy alone, and EBRT combined with brachytherapy [2]. EBRT combined with a high dose-rate brachytherapy (HDR-BT) boost is a long-established method of radiotherapy dose escalation [3–5]. Irrespective of the chosen treatment paradigm, patients usually face a favorable prognosis living for many years after diagnosis [6]. This underlines the importance of survivorship issues including decision regret after a chosen treatment.

Decision regret has been defined in a concept analysis by Chehade and colleagues as “*a negative cognitive-emotional phenomenon experienced following a health-related decision in an immediate or delayed occurrence*” [7]. Decision regret is common after health-care decisions. Rühle and colleagues showed that approximately 57% of patients with various types of cancer reported at least some degree of decision regret after radiotherapy as reported on the patient-reported outcome measure Decision Regret Scale (DRS) [8, 9]. On a scale ranging from 0 to 100 with higher scores indicating worse regret, the mean score was 14 as reported in this study. A systematic review on decision regret across health conditions reported a comparable mean score of 17 [10]. Further, Chehade and colleagues define that “*regret relates to the decision-making process, treatment options, and/or outcomes and results in negative and positive consequences*” [7]. Accordingly, various determinants of decision regret have been reported. These include health-related quality of life (HRQoL), shared decision-making, the balance of expected and experienced results, and even in one publication on head and neck cancer patients dosimetric parameters such as radiation dose per fraction [10–12]. However, to our knowledge, no study has elucidated the extent and determinants of decision regret after EBRT combined with HDR-BT boost for prostate cancer.

Therefore, we undertook a secondary analysis of a preregistered cross-sectional study of patients treated with EBRT combined with HDR-BT boost for prostate cancer. Our co-primary objectives were, first, to describe the prevalence of decision regret. Second, we aimed to investigate determinants of decision regret including patient characteristics, dose to organs at risk (OAR), and patient-reported outcomes.

## Materials and methods

### Study design

This is a secondary *post-hoc* analysis of a protocol-based (doi.org/10.17605/OSF.IO/A6DC3) cross-sectional study conducted at an academic tertiary cancer center in Germany. The primary outcome of our study and study methods have been reported previously [13]. In brief, ethical approval was obtained prior to enrolment of the first patient. Patients were eligible if they (i) had histologically confirmed prostate cancer, (ii) had EBRT + HDR-BT boost as primary treatment at least two years ago, (iii) had no evidence of disease per Phoenix-criteria (PSA > 2 ng/ml above nadir), (iv) had no foley catheter at the time of the survey, (v) had no surgical intervention to the genitourinary tract after radiation, (vi) were able to understand and self-report questionnaires, (vii) were older than 18 years, and (viii) gave written informed consent. We contacted potentially eligible patients consecutively. Consenting patients then received and returned patient-reported outcomes via post mail. Only patients with given information on decision regret were eligible for the present analysis. The STROBE guideline was respected for reporting the study in this and the previous report as applicable [13, 14].

### Treatment

The prostate, seminal vesicles, and typically lymph nodes in the small pelvis were treated in 2 Gy per fraction up to a total dose of 40–50 Gy in 20–25 fractions in 5 weekly fractions of EBRT. During the EBRT course, two fractions of ultrasound-guided HDR-BT boost were delivered two weeks apart. The whole prostate gland received 8 Gy and the peripheral zone of the prostate 15 Gy at each of both HDR-BT fractions [5].

### Outcomes and variables

Patient-reported outcome measures included following validated questionnaires: DRS, Expanded Prostate Cancer Indec Composite (EPIC- 26), EORTC QLQ-C30, Patient Satisfaction with Cancer-related Care (PSCC), and Self-Administered Comorbidity Questionnaire (SCQ) [9, 15–19].

The focus of the present analysis are results of decision regret per DRS. Patients were asked to complete the DRS in light of the received radiotherapy. The DRS includes 5 items patients respond to in a 5-point likert scaled fashion: (i.) “It was the right decision”, (ii.) “I regret the choice that was made”, (iii.) “I would go for the same choice if I had to do it over again”, (iv.) “The choice did me a lot of harm”, and (v.) “The decision was a wise one”. To evaluate DRS results, scores of both negatively framed items (item two and four) were reversed. Responses to each item ranging from 1 to 5 were subtracted by 1 and multiplied by 25. Then a summary score ranging from 0 to 100 was calculated from the mean of all five items. Higher values indicate higher levels of regret. We categorized levels of regret as previously described in “no decision regret” (0 points), “mild decision regret” (1–25 points), and “strong decision regret” (> 25 points) [8, 9].

The EPIC- 26 questionnaire includes five HRQoL domain summary scores concerning patients with prostate cancer: urinary incontinence, urinary irritative/obstructive, bowel (“overall gastrointestinal function”), sexual, and hormonal. It also includes a single question on overall urinary function. Higher values indicate better functioning. The EORTC QLQ-C30 is a generic measure to evaluate HRQoL of patients with cancer. We hypothesized that four domains might interact with decision regret based on published literature: (i.) physical functioning, (ii) emotional functioning, (iii.) pain, and (iv.) fatigue [20]. Higher scores of physical and emotional functioning indicate better function, whereas higher scores of pain and fatigue indicate worse symptoms.

The PSCC questionnaire includes 18 single item questions on patient satisfaction [18]. Of these, we used “question 3” as indicator for the level of shared decision making: “I felt included in decisions about my health”. We also used a PSCC total score across all 18 items for overall patient satisfaction. Patients answer PSCC questions on a 5-point likert scale and normalized values range from 0 to 100 with higher values indicating a higher level of shared decision making or satisfaction, respectively.

Dose exposure to OAR was evaluated for the urethra, bladder neck, and rectum as previously described [13]. In brief, we evaluated urethral doses of D_Urethra_0.1 cc, D_Urethra_10%, and D_Urethra_30%. The dose to the bladder neck was evaluated as maximum point dose. Dose levels to the rectum included D_Rectum_0.1 cc, D_Rectum_1cc, and D_Rectum_2cc. All dose exposures were calculated as summary values comprising the EBRT dose and HDR-BT dose as previously described [13].

Additional medical data and patient characteristics were retrieved from medical records and from updated medical histories.

### Statistical analysis

We used descriptive statistics to display the study cohort and the distribution of decision regret. To examine associations of decision regret and covariables, we employed Pearson’s correlation for continuous and one-way ANOVA for non-continuous data. In light of a left-skewed decision regret mean score (skewness = 1.2) and the given scope of our data set, we decided not to perform a multivariable linear regression model of decision regret [21]. We did not correct for multiple testing due to the exploratory nature of our study [22]. A two-sided p-value of < 0.05 was considered statistically significant. All analyses were performed with JASP v0.18.3 (JASP Team [2022], Amsterdam, the Netherlands).

## Results

### Characteristics of the study cohort

Among 125 participating patients, 108 (86%) completed the DRS as shown in the flow chart in Supplementary Fig. 1. All patients with available responses to the DRS were included in further analyses. Patient characteristics are shown in Table 1.

**Table 1:** Patient (n = 108) and treatment characteristics

Patient characteristics		
Age at radiotherapy [years]		Median: 73; IQR: 8
Age at survey [years]		Median: 77; IQR: 9
Performance status at radiotherapy	ECOG 0	90% (97)
	ECOG 1	10% (11)
D’Amico risk group at radiotherapy	Low risk	5% (6)
	Intermediate risk	57% (62)
	High risk	38% (40)
Prostate volume at radiotherapy [mL]		Mean: 33; SD: 10
History of TURP prior to radiotherapy [Yes]		10% (12)
History of smoking at survey [Yes]		62% (70)
Use of antiobstructive medication at survey [Yes]		37% (41)
Number of comorbidities at survey		Median: 2, IQR: 2
History of depression at survey [Yes]		5% (5)
Global health status/QoL at survey [EORTC QLQ-C30]		Mean: 74; SD: 18
**Treatment characteristics**		
EBRT technique	3DCRT	71% (77)
	IMRT/VMAT	29% (31)
Number of needles in HDR-BT		Median: 14; IQR: 3
History of ADT [Yes]		40% (43)
D_Urethra_0.1 cc [Gy]^#^		Mean: 114; SD: 14
D_Uretha_10% [Gy]^#^		Mean: 112; SD: 10
D_Urethra_30% [Gy]^#^		Mean: 101; SD: 10
D_Bladderneck_max [Gy]^#^		Mean: 71; SD: 7
D_Rectum_0.1 cc [Gy]^#^		Mean: 107; SD: 13
D_Rectum_1cc [Gy]^#^		Mean: 89; SD: 10
D_Rectum_2cc [Gy]^#^		Mean: 79; SD: 9

Absolute numbers are given in brackets. Numbers may not add up to 100% due to rounding error or missing values. Global health status/QoL: higher score indicates better health-related quality of life

*ADT* androgen deprivation therapy, *EBRT* external beam radiation therapy, *HDR-BT* high dose rate brachy therapy, *QoL* quality of life, *IMRT* intensity modulated radiation therapy, *IQR* interquartile range, *PSA* prostate specific antigen, *SD* standard deviation, *TURP* transurethral resection of the prostate, *VMAT* volume modulated arc therapy, *3DCRT* 3D conformal radiotherapy

^#^Cumulative equivalent doses in 2 Gy per fraction (EQD2) of EBRT (prescribed total dose) and HDR-BT (actual doses based on ultrasound planning of each fraction) plans. Alpha/Beta-Ratios were 1.5 for the urethra as well as bladder neck and 2.5 for the rectum

### Prevalence of decision regret

**Fig. 1:**
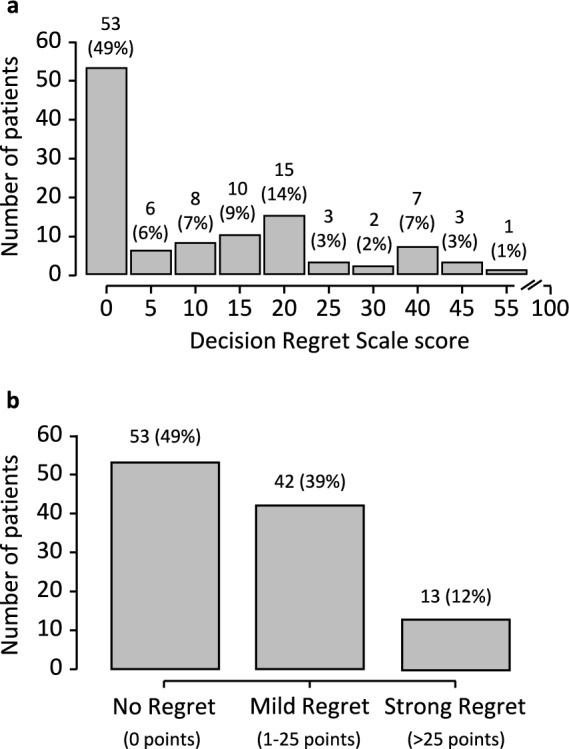
Distribution of decision regret among prostate cancer patients (n = 108) at least two years after external beam radiotherapy and HDR-brachytherapy boost according to the Decision Regret Scale questionnaire. Panel a shows a histogram. The maximum value in our cohort was 55 points. Panel b shows the categorial distribution of no (0 points), mild (1–25 points), or strong (> 25 points) regret. Absolute numbers are shown and percentages are given in brackets

The distribution of decision regret based on the DRS is shown in Fig. 1. Patients reported a median value of 5 (IQR: 20) of decision regret. The mean value of decision regret was 11 (SD: 14). The maximum value was 55 points. No, mild, or strong decision regret was present in 49% (53/108), 39% (42/108), and 12% (13/108) of the patients, respectively. Scores of single items of the DRS are shown in Supplementary Table 1.

### Associations of general patient characteristics and decision regret

We did not observe statistically significant associations of general patient characteristics and decision regret (Supplementary Table 2 and Table 2). These general patient characteristics included performance status (ECOG), d’Amico risk group, history of TURP, use of antiobstructive prostate medication, history of ADT, history of depression, age at radiotherapy, time passed since radiotherapy, prostate volume at radiotherapy and, number of comorbidities.

**Table 2:** Association of decision regret per Decision Regret Scale and continuous independent variables per Pearson’s correlation

Decision regret	N	Pearson’s r	p	Lower 95% CI	Upper 95% CI
Age at radiotherapy [years]	108	− 0.131	0.178	− 0.312	0.060
Time passed since radiotherapy [months]	108	− 0.119	0.222	− 0.301	0.072
Prostate volume at radiotherapy [mL]	108	− 0.002	0.982	− 0.191	0.187
Number of comorbidities at survey	108	0.026	0.792	− 0.164	0.214
D_Urethra_0.1 cc [Gy]	108	0.037	0.703	− 0.153	0.224
D_Uretha_10% [Gy]	108	0.071	0.465	− 0.119	0.257
D_Urethra_30% [Gy]	108	0.095	0.326	− 0.095	0.279
D_Bladderneck_max [Gy]	108	0.039	0.686	− 0.151	0.227
D_Rectum_0.1 cc [Gy]	108	− 0.006	0.951	− 0.195	0.183
D_Rectum_1cc [Gy]	108	0.095	0.326	− 0.095	0.279
D_Rectum_2cc [Gy]	108	0.095	0.326	− 0.095	0.279
Urinary incontinence—EPIC 26	102	− 0.198	**0.046**	− 0.378	− 0.004
Urinary irritative/obstructive—EPIC 26	98	− 0.203	**0.045**	− 0.386	− 0.005
Urinary overall function—EPIC 26	107	− 0.070	0.474	− 0.256	0.121
Bowel function—EPIC 26	103	− 0.312	**0.001**	− 0.477	− 0.126
Hormonal function—EPIC 26	103	− 0.289	**0.003**	− 0.457	− 0.101
Sexual function—EPIC 26	105	− 0.162	0.099	− 0.343	0.031
Physical functioning—EORTC QLQ-C30	106	− 0.111	0.256	− 0.296	0.081
Emotional functioning – EORTC QLQ-C30	104	− 0.075	0.450	− 0.264	0.119
Pain—EORTC QLQ-C30	103	0.022	0.826	− 0.172	0.215
Fatigue—EORTC QLQ-C30	104	0.098	0.322	− 0.096	0.285
Shared decision making—PSCC	107	− 0.292	**0.002**	− 0.456	− 0.108
Patient satisfaction—PSCC	103	− 0.326	** < 0.001**	− 0.489	− 0.141

Bold text represents statistically significant p-values < 0.05

*EPIC- 26* Expanded prostate cancer index composite, *EORTC QLQ-C30* European Organization for Research and Treatment of Cancer Quality of Life Core Questionnaire, *PSCC* Patient Satisfaction with Cancer-related Care

### Associations of dose to organs at risk and decision regret

Cumulative radiation dose exposure levels to the OARs urethra, bladder neck, and rectum are shown in Table 1. There were no statistically significant associations of OAR dose levels and decision regret (Table 2).

### Associations of patient-reported outcomes and decision regret

Patient-reported HRQoL of relevant domains of the EPIC- 26 and EORTC QLQ-C30 is shown in Supplementary Table 3. There was a statistically significant association of higher decision regret and worse EPIC- 26 urinary incontinence (Pearson’s r = − 0.198; 95%-CI: − 0.378 to − 0.004; p = 0.046), worse EPIC- 26 urinary irritative/obstructive symptoms (Pearson’s r = 0.203; 95%-CI: − 0.386 to − 0.005; p = 0.045), worse EPIC- 26 bowel function (Pearson’s r = − 0.312; 95%-CI: − 0.477 to − 0.126; p = 0.001), and worse EPIC- 26 hormonal function (Pearson’s r = − 0.289; 95%-CI: − 0.457 to − 0.101; p = 0.003), respectively (Table 2). Further HRQoL domains including EPIC- 26 sexual function, EORTC QLQ-C30 physical functioning, EORTC QLQ-C30 emotional functioning, EORTC QLQ-C30 pain, and EORTC QLQ-C30 fatigue were not significantly associated with decision regret.

Concerning shared-decision making, patients reported a mean value of 94 (SD: 12) based on question 3 of the PSCC. We found a statistically significant association of lower decision regret with higher levels of shared decision making (Pearson’s r = − 0.292; 95%-CI: − 0.456 to − 0.108; p = 0.002) (Table 2). Overall patient satisfaction was reported at a mean value of 84 (SD: 8) based on the PSCC total score. There was a statistically significant association of lower decision regret with higher patient satisfaction (Pearson’s r = − 0.326; 95%-CI: − 0.489 to − 0.141; p < 0.001) (Table 2).

## Discussion

This secondary analysis of a cross-sectional study demonstrated a moderate extent of decision regret and its associations with patient-reported outcomes in patients treated with EBRT and HDR-BT boost for prostate cancer.

### Prevalence of decision regret

Other studies have reported on the prevalence of decision regret using the DRS in varying clinical scenarios. Wilding and colleagues, for example, conducted a cross-sectional study among more than 17,000 patients with localized prostate cancer in the UK [23]. This cohort included patients managed with EBRT, surgery or monitoring at 18 months after diagnosis. Herein, 37% of the patients reported no decision regret, 43% mild decision regret and 20% strong decision regret. Another study surveyed 434 patients with prostate cancer in the Netherlands undergoing either EBRT, brachytherapy alone, surgery, or active surveillance [20]. At 12 months after treatment, 23% of the patients expressed strong regret by analogy to the regret categories in our analysis. The type of treatment was not associated with decision regret in this analysis. Furthermore, Rühle and colleagues conducted a cross-sectional study among 207 patients with various types of cancer treated with radiotherapy in Germany [8]. In this study, 43% of the patients reported no decision regret, 38% mild decision regret and 18% strong decision regret. In light of the published literature, the prevalence of decision regret compares favorably in our cohort as 49% of the patients reported no decision regret, 39% mild decision regret and only 12% strong decision regret.

### Determinants of decision regret

Regarding determinants of decision regret, we did not observe associations of general patient characteristic such as a patient’s age. This finding is supported by a recent systematic review of decision regret among prostate cancer patients that did not report a consistent impact of age on decision regret across studies [11]. However, we found associations of patient-reported outcomes with decision regret and this is again found in published literature of various clinical scenarios. The negative impact of worse HRQoL and its subdomains including bowel, urinary, and hormonal symptoms on decision regret has repeatedly been demonstrated in cohort studies as well as systematic reviews [10, 11, 20, 24–27]. Similarly, earlier studies have reported on the impact of higher levels of shared decision making and patient satisfaction on lower levels of decision regret. This is the case for patients with prostate cancer as well as patients with various types of cancer [8, 11, 24, 28, 29]. These studies on the interplay of patient-reported outcomes and decisional regret mainly analyzed patients undergoing EBRT, active surveillance, surgery or mono brachytherapy but not EBRT and HDR-BT boost. Taken together, the associations of patient-reported outcomes in terms of HRQoL and shared-decision making as well as patient satisfaction are consistent with the literature of other clinical settings. To potentially limit decision regret, these factors should be respected along with patient preferences prior to treatment. For example, an open discussion before therapy about specific side effects associated with each treatment option can help patients to weigh potential morbidity in light of their personal preferences. Further, decision aids may help to support in this process of shared-decision making [30].

### Limitations

Limitations to our analysis include, first, that our cohort does not include patients with recurrent disease. Although the published literature is unambiguous on this issue, recurrent disease could have led to higher decision regret [8, 20, 24]. Second, we purposefully did not conduct a multivariable analysis in light of uncertainties concerning variable selection and generalizability based on this secondary post hoc analysis. Even though in line with the published literature, our results could therefore be confirmed by a multivariable analysis in a larger prospective multicenter study. Third, although we found plausible associations of patient-reported outcomes and decision regret, effect sizes were weak or moderate.

## Conclusions

Decision regret appears relatively mild in a cohort of patients treated with EBRT and HDR-BT boost for prostate cancer. Patient-reported outcomes, including HRQoL, shared-decision making, and patient satisfaction, were associated with decision regret which should be respected in clinical practice.

## Supplementary Information

Below is the link to the electronic supplementary material.

Supplementary file1 (PDF 153 KB)

## Data Availability

No datasets were generated or analysed during the current study.

## Abbreviations

ADT: Androgen deprivation therapy
DRS: Decision Regret Scale
EBRT: External beam radiotherapy
EPIC- 26: Expanded Prostate Cancer Indec Composite
EQD2: Equivalent doses in 2 Gy per fraction
HDR-BT: High dose-rate brachytherapy
HRQoL: Health-related quality of life
IPSS: International Prostate Symptom Score
OAR: Organs at risk
PSCC: Patient satisfaction with cancer-related care
SBRT: Stereotactic body radiotherapy
SCQ: Self-Administered Comorbidity Questionnaire
TURP: Transurethral resection of the prostate

## References

[1] Bray F, Laversanne M, Sung H et al (2024) Global cancer statistics 2022: GLOBOCAN estimates of incidence and mortality worldwide for 36 cancers in 185 countries. CA A Cancer J Clin. 74:229–263. 10.3322/caac.21834 10.3322/caac.21834 38572751

[2] Rebello RJ, Oing C, Knudsen KE et al (2021) Prostate cancer. Nat Rev Dis Primers 7:1–27. 10.1038/s41572-020-00243-0 33542230 10.1038/s41572-020-00243-0

[3] Nikitas J, Kishan A, Chang A et al (2024) Treatment intensification strategies for men undergoing definitive radiotherapy for high-risk prostate cancer. World J Urol 42:165. 10.1007/s00345-024-04862-0 38492111 10.1007/s00345-024-04862-0

[4] Galalae RM, Zakikhany NH, Geiger F et al (2014) The 15-year outcomes of high-dose-rate brachytherapy for radical dose escalation in patients with prostate cancer—a benchmark for high-tech external beam radiotherapy alone? Brachytherapy 13:117–122. 10.1016/j.brachy.2013.11.002 24360880 10.1016/j.brachy.2013.11.002

[5] Siebert F-A, Wolf S, Bertermann H et al (2014) Introduction of inverse dose optimization for ultrasound-based high-dose-rate boost brachytherapy: how we do it in Kiel. Brachytherapy 13:250–256. 10.1016/j.brachy.2014.01.010 24613132 10.1016/j.brachy.2014.01.010

[6] Hamdy FC, Donovan JL, Lane JA et al (2023) Fifteen-year outcomes after monitoring, surgery, or radiotherapy for prostate cancer. N Engl J Med. 10.1056/NEJMoa2214122 36912538 10.1056/NEJMoa2214122

[7] Chehade M, Mccarthy MM, Squires A (2024) Patient-related decisional regret: an evolutionary concept analysis. J Clin Nurs 33:4484–4503. 10.1111/jocn.17217 38757768 10.1111/jocn.17217

[8] Rühle A, Wieland L, Hinz A et al (2024) Decision regret of cancer patients after radiotherapy: results from a cross-sectional observational study at a large tertiary cancer center in Germany. J Cancer Res Clin Oncol 150:167. 10.1007/s00432-024-05638-0 38546873 10.1007/s00432-024-05638-0 PMC10978708

[9] Brehaut JC, O’Connor AM, Wood TJ et al (2003) Validation of a decision regret scale. Med Decis Making 23:281–292. 10.1177/0272989X03256005 12926578 10.1177/0272989X03256005

[10] Becerra Pérez MM, Menear M, Brehaut JC, Légaré F (2016) Extent and predictors of decision regret about health care decisions: a systematic review. Med Decis Making 36:777–790. 10.1177/0272989X16636113 26975351 10.1177/0272989X16636113

[11] Gartrell BA, Phalguni A, Bajko P et al (2024) Influential factors impacting treatment decision-making and decision regret in patients with localized or locally advanced prostate cancer: a systematic literature review. Eur Urol Oncol S2588–9311(24):00106–00108. 10.1016/j.euo.2024.04.016 10.1016/j.euo.2024.04.016 38744587

[12] Köksal M, Saur L, Scafa D et al (2022) Late toxicity-related symptoms and fraction dose affect decision regret among patients receiving adjuvant radiotherapy for head and neck cancer. Head Neck 44:1885–1895. 10.1002/hed.27103 35635498 10.1002/hed.27103

[13] Haack L, Krug D, Domschikowski J et al (2025) Associations of dose to the urethra and long-term patient-reported outcomes after radiotherapy with EBRT and HDR brachytherapy boost for prostate cancer. ctRO. 10.1016/j.ctro.2025.100918 39898332 10.1016/j.ctro.2025.100918 PMC11782951

[14] von Elm E, Altman DG, Egger M et al (2008) The Strengthening the Reporting of Observational Studies in Epidemiology (STROBE) statement: guidelines for reporting observational studies. J Clin Epidemiol 61:344–349. 10.1016/j.jclinepi.2007.11.008 18313558 10.1016/j.jclinepi.2007.11.008

[15] Beyer B, Huland H, Feick G, Graefen M (2015) Expanded prostate cancer index composite (EPIC-26). Urologe 54:1591–1595. 10.1007/s00120-015-3922-0 10.1007/s00120-015-3922-0 26347350

[16] Sibert NT, Dieng S, Oesterle A et al (2021) Psychometric validation of the German version of the EPIC-26 questionnaire for patients with localized and locally advanced prostate cancer. World J Urol 39:11–25. 10.1007/s00345-019-02949-7 31552467 10.1007/s00345-019-02949-7

[17] Aaronson NK, Ahmedzai S, Bergman B et al (1993) The European Organization for Research and Treatment of Cancer QLQ-C30: a quality-of-life instrument for use in international clinical trials in oncology. J Natl Cancer Inst 85:365–376. 10.1093/jnci/85.5.365 8433390 10.1093/jnci/85.5.365

[18] Bokemeyer F, Lange-Drenth L, Jean-Pierre P et al (2020) Psychometric evaluation of the German version of the Patient Satisfaction with Cancer-related Care questionnaire. BMC Health Serv Res 20:983. 10.1186/s12913-020-05838-7 33109191 10.1186/s12913-020-05838-7 PMC7590742

[19] Sangha O, Stucki G, Liang MH et al (2003) The Self-Administered Comorbidity Questionnaire: a new method to assess comorbidity for clinical and health services research. Arthritis Rheum 49:156–163. 10.1002/art.10993 12687505 10.1002/art.10993

[20] van Stam M-A, Aaronson NK, Bosch JLHR et al (2020) Patient-reported outcomes following treatment of localised prostate cancer and their association with regret about treatment choices. Eur Urol Oncol 3:21–31. 10.1016/j.euo.2018.12.004 31411965 10.1016/j.euo.2018.12.004

[21] Greenland S, Senn SJ, Rothman KJ et al (2016) Statistical tests, P values, confidence intervals, and power: a guide to misinterpretations. Eur J Epidemiol 31:337–350. 10.1007/s10654-016-0149-3 27209009 10.1007/s10654-016-0149-3 PMC4877414

[22] Bender R, Lange S (2001) Adjusting for multiple testing—when and how? J Clin Epidemiol 54:343–349. 10.1016/S0895-4356(00)00314-0 11297884 10.1016/s0895-4356(00)00314-0

[23] Wilding S, Downing A, Selby P et al (2020) Decision regret in men living with and beyond nonmetastatic prostate cancer in the United Kingdom: a population-based patient-reported outcome study. Psychooncology 29:886–893. 10.1002/pon.5362 32065691 10.1002/pon.5362 PMC7317932

[24] Lunger L, Meissner VH, Kopp BCG et al (2023) Prevalence and determinants of decision regret in long-term prostate cancer survivors following radical prostatectomy. BMC Urol 23:139. 10.1186/s12894-023-01311-9 37612591 10.1186/s12894-023-01311-9 PMC10464370

[25] Hoffman RM, Lo M, Clark JA et al (2017) Treatment decision regret among long-term survivors of localized prostate cancer: results from the prostate cancer outcomes study. J Clin Oncol 35:2306–2314. 10.1200/JCO.2016.70.6317 28493812 10.1200/JCO.2016.70.6317 PMC5501361

[26] Shaverdian N, Verruttipong D, Wang P-C et al (2017) Exploring value from the patient’s perspective between modern radiation therapy modalities for localized prostate cancer. Int J Radiat Oncol Biol Phys 97:516–525. 10.1016/j.ijrobp.2016.11.007 28126301 10.1016/j.ijrobp.2016.11.007

[27] Hilger C, Schostak M, Otto I, Kendel F (2021) Time pressure predicts decisional regret in men with localized prostate cancer: data from a longitudinal multicenter study. World J Urol 39:3755–3761. 10.1007/s00345-021-03727-0 34021406 10.1007/s00345-021-03727-0 PMC8519821

[28] Skyring TA, Mansfield KJ, Mullan JR (2021) Factors affecting satisfaction with the decision-making process and decision regret for men with a new diagnosis of prostate cancer. Am J Mens Health 15:15579883211026812. 10.1177/15579883211026812 34261353 10.1177/15579883211026812 PMC8287369

[29] Sato M, Osawa T, Nishioka K et al (2024) Decision regret after curative treatment and its association with the decision-making process and quality of life for prostate cancer patients. Int J Urol. 10.1111/iju.15602 39382251 10.1111/iju.15602

[30] Lamers RED, Cuypers M, de Vries M et al (2021) Differences in treatment choices between prostate cancer patients using a decision aid and patients receiving care as usual: results from a randomized controlled trial. World J Urol 39:4327–4333. 10.1007/s00345-021-03782-7 34272972 10.1007/s00345-021-03782-7 PMC8602175

