# Supplementary material for: Decision regret after external beam radiotherapy and high dose-rate brachytherapy boost for prostate cancer
Source: World J Urol. 2025 Apr 9;43(1):220. doi: 10.1007/s00345-025-05615-3 (PMC11982119; doi:10.1007/s00345-025-05615-3)
Supplement: Supplementary file 1 — Supplementary file1 (PDF 153 KB) [file 345_2025_5615_MOESM1_ESM.pdf]

Decision regret after external beam radiotherapy and HDR-brachytherapy boost for prostate cancer

Lars Haack[1], David Krug [1,2], Severin Rodler[3], Philipp Nuhn[3], Christof van der Horst[4], Christian Schulz[1], Olaf Wittenstein[1], Claudia Schmalz[1], Oliver Blanck[1], Frank-André Siebert[1], Alexander Fabian[1 #]

# Corresponding author: Alexander Fabian, MD (alexander.fabian@uksh.de)
Department of Radiation Oncology, University Hospital Schleswig-Holstein Campus Kiel, Kiel, Germany

Supplementary Figure 1 Flow chart of participating and analyzed patients (n=108). Abbreviation: RT radiotherapy

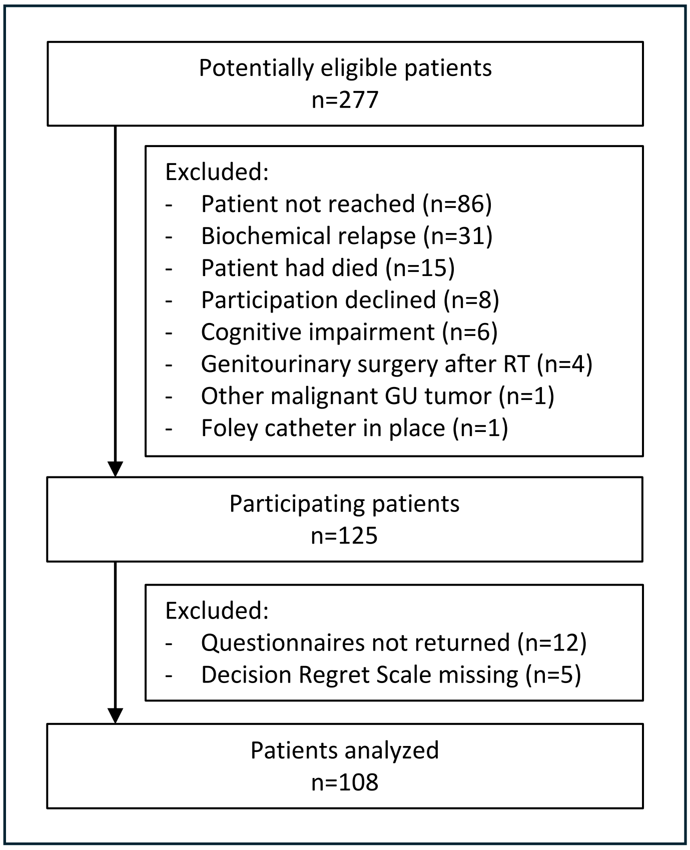

Supplementary Table 1 Results of single items of the Decision Regret Scale (n=108). A higher mean on a single item indicates higher agreement, Range 0-100. A higher mean on the total score indicates higher decision regret, Range 0-100. Abbreviations: SD Standard deviation.

Item No.	Item	Mean	SD	Range
1	It was the right decision	2	9	0-50
2	I regret the choice that was made	10	26	0-100
3	I would go for the same choice if I had to do it over again	6	18	0-100
4	The choice did me a lot of harm	28	36	0-100
5	The decision was a wise one	8	22	0-100
	Total Score	11	14	0-55

Supplementary Table 2 Association of decision regret per Decision Regret Scale and non-continuous independent variables per one-way ANOVA (n=108). Abbreviations: ADT, androgen deprivation therapy ; SD, standard deviation; TURP, transurethral resection of the prostate

Variable	N	Mean	SD	p
Performance status at radiotherapy				0.102
ECOG 0	97	10.1	13.7	
ECOG 1	11	17.3	14.6	
D'Amico Risk Group				0.590
Low Risk	6	12.5	17.8	
Intermediate Risk	62	11.8	14.7	
High Risk	40	9.0	12.1	
History of TURP				0.836
Yes	12	10.0	16.2	
No	96	10.9	13.7	
Use of antiobstructive prostate medication				0.969
Yes	41	10.9	14.8	
No	67	10.8	13.4	
History of ADT				0.634
Yes	43	10.0	13.4	
No	65	11.4	14.3	
History of depression				0.972
Yes	5	11	10.8	
No	103	10.8	14.0	

Supplementary Table 3 Selected patient-reported outcomes (n = 108). EPIC-26 domains show better functioning with higher scores. EORTC QLQ-C30 domains show better functioning with higher function scores, but worse symptoms with higher symptom scores. Abbreviations: EPIC-26, Expanded prostate cancer index composite; EORTC QLQ-C30, European Organization for Research and Treatment of Cancer Quality of Life Core Questionnaire; PSCC, Patient Satisfaction with Cancer-related Care

Domain	Questionnaire	Mean	Standard deviation
Urinary incontinence	EPIC-26	86	22
Urinary irritative/obstructive	EPIC-26	90	12
Urinary overall function	EPIC-26	81	25
Bowel function	EPIC-26	93	14
Hormonal function	EPIC-26	89	16
Sexual function	EPIC-26	31	23
Physical functioning	EORTC QLQ-C30	81	20
Emotional functioning	EORTC QLQ-C30	87	17
Pain	EORTC QLQ-C30	18	23
Fatigue	EORTC QLQ-C30	24	24
